# Low intensity, long-term outpatient rehabilitation in COPD: a randomised controlled trial

**DOI:** 10.1186/1465-9921-13-86

**Published:** 2012-09-27

**Authors:** Hans Jörg Baumann, Stefan Kluge, Katrin Rummel, Hans Klose, Jan K Hennigs, Tibor Schmoller, Andreas Meyer

**Affiliations:** 1 Department of Respiratory Medicine, University Medical Center Hamburg-Eppendorf, Hamburg, Germany; 2 Department of Intensive Care Medicine, University Medical Center Hamburg-Eppendorf, Hamburg, Germany; 3 Pulmonary practice Winterhude, Hamburg, Germany; 4 Kliniken Mariahilf GmbH, Department of Pneumology, Mönchengladbach, Germany

**Keywords:** COPD, Exercise training, Pulmonary rehabilitation, Quality of life

## Abstract

**Background:**

Most pulmonary rehabilitation programmes currently involve 2–3 sessions per week as recommended by international guidelines. We aimed to investigate whether relevant improvements in physical capabilities and quality of life in patients with chronic obstructive pulmonary disease (COPD) could be achieved by a long-term, low intensity, once weekly rehabilitation programme using limited resources.

**Methods:**

100 patients with moderate to severe COPD were randomised to a continuous outpatient interdisciplinary rehabilitation programme or standard care. Physiotherapy-led supervised outpatient training sessions were performed once weekly in addition to educational elements. Outcome measures at baseline and after 26 weeks were 6-minute-walk-test, cycle ergometry, and health-related quality of life.

**Results:**

37 patients in the training group and 44 patients in the control group completed the study. After 26 weeks there were clinically significant differences between the groups for 6 minute-walk-distance (+59 m, 95% CI 28–89 m), maximum work load (+7.4 Watt, 95% CI 0.5-13.4 Watt) and St. George’s Respiratory Questionnaire score (−5 points, 95% CI −10 to −1 points). Total staff costs of the programme per participant were ≤ €625.

**Conclusion:**

Clinically meaningful improvements in physical capabilities and health-related quality of life may be achieved using long-term pulmonary rehabilitation programmes of lower intensity than currently recommended. Trial registration: clinicaltrials.gov NCT01195402.

## Introduction

There is considerable evidence of the beneficial effects of pulmonary rehabilitation on exercise capabilities and health-related quality of life (HRQoL) in patients with chronic obstructive pulmonary disease (COPD) [1]. Clinically relevant effects may be achieved by rehabilitation programmes of differing designs in terms of setting (inpatient vs. outpatient vs. home-based), duration (short-term vs. long-term), and intensity (high vs. low intensity) [1]. As it is well known that achieved improvements decline following short-term programmes, efforts have been made to improve long-term maintenance [1].

Although the evidence for the efficacy of pulmonary rehabilitation is strong and it is highly recommended by current guidelines, only a minority of eligible COPD patients is included in rehabilitation programmes [2]. Reasons for this discrepancy may be lack of belief in the efficacy of such programmes, lack of local access, and concerns about the cost. While the first aspect should be addressed by intensified promotion on the beneficial effects of pulmonary rehabilitation in the medical community, the other two may be answered by the design of simple and locally available programmes using a minimum amount of resources that still produce clinically relevant effects.

Current guidelines on pulmonary rehabilitation recommend interventions with a frequency of at least 2–3 supervised sessions of high-intensity training per week [1]. This facilitates optimal short-term results, but may lead to lower adherence rates and higher costs in the long-term.

Ambulatory ‘lung sport’ groups with 15–20 participants and weekly sessions, which are fairly common in Germany, have shown long-term effects on cardiopulmonary fitness in mild to moderate obstructive airways diseases [3,4]. However, patients with more severe limitations may find it difficult to exercise with those with less severe disease. Therefore, we developed the concept of physiotherapist-lead training groups of 6–10 members where it is possible to tailor training intensity to the different, and often very limited, physical capabilities of the participants.

The purpose of the present study was to evaluate whether a continuous, low-intensity, long-term, physiotherapist-lead outpatient pulmonary rehabilitation programme can induce significant improvements in the exercise capabilities and HRQoL in patients with moderate to severe COPD using less resources than previously published programmes.

## Material and methods

### Study design

The study was a prospective, randomised, controlled, interventional, multicentre trial involving six local physiotherapy practices. Randomisation was performed using a computer-generated list of random numbers to assign participants to either training or standard care. Due to the nature of the intervention it was not possible to blind subjects to their allocation. The study was scheduled to last for 26 weeks. Only subjects with both baseline and 6-month measurements were included in the secondary analyses. No attempt was made to impute missing data.

Ethics approval was obtained from the local ethics committee. Approval covered the protocol and consent form used to obtain written informed consent from all subjects. The study was registered with ClinicalTrials.gov: NCT01195402.

### Subjects

Consecutive patients with COPD according to accepted criteria [5] were recruited from 14 pulmonary specialist practices in the Hamburg metropolitan area. Inclusion criteria were: 1) age between 50 and 80 years, 2) COPD GOLD stages II-IV, 3) smoking history of >20 pack years, 4) pharmacological therapy according to the current guidelines, 5) written informed consent. Exclusion criteria were 1) respiratory insufficiency, defined as PaO_2_ <55 mm Hg and/or PaCO_2_ >50 mm Hg breathing room air, 2) manifest cardiac insufficiency, 3) uncontrolled arterial hypertension, 4) active malignant disease, 5) symptomatic coronary heart disease or pathological test results in cycle ergometry, 6) limited physical capabilities caused by musculoskeletal disorders, 7) unwillingness to return for follow-up, 8) previous or ongoing participation in exercise training programmes, and 9) expected inability to attend at least 75% of sessions. The patient‘s pulmonologists assessed the patients regarding the ability to participate safely in the exercise training. Eligible patients were provided with details regarding the study. Following informed consent they were included into the study. Medication was managed at the discretion of the subject’s pulmonary specialist in both groups.

### Assessments

Outcome measures were obtained following randomisation at the beginning of the study and after a period of 6 months. Exercise tolerance was measured using the 6-minute walk test (6MWT) and cycle ergometry. The 6MWT constituted the primary outcome measure, while maximum work load, maximum oxygen uptake and HRQoL assessements were the secondary outcomes.

#### Physiological measures

Pulmonary function tests using body plethysmography were done by the patients’ pulmonary specialists according to accepted methods [6]. Using a standardised protocol, 6MWT was performed twice to avoid learning effects [7]. Results were compared to published reference values [8]. The 6MWT in the training group was done in the physiotherapy practices whereas the control group was tested in the University Medical Center Hamburg-Eppendorf. The staff performing the 6MWT was therefore not blinded to allocation status.

Cycle ergometry for the incremental exercise test was done either at the University Medical Center Hamburg-Eppendorf or in one of three selected pulmonary specialists’ practices according to standard guidelines using a ramp protocol (+10 Watt/min starting at 0 Watt) until exhaustion [9]. Assessors were blinded to the randomisation status.

#### Psychosocial measures

HRQoL was assessed using German versions of the Short Form-12 (SF-12) questionnaire as a general instrument and the St. George’s Respiratory Questionnaire (SGRQ) [10] as a disease specific instrument. The SF-12 is self-administered, incorporates 12 items and yields 2 separate subscales: the Physical Component Summary Score and the Mental Health Component Summary Score [11,12].

#### Control group

Standard care consisted of referral back to the participant’s pulmonologist following baseline assessments. The control group did not take part in any components of the rehabilitation programme.

#### Training group

The interdisciplinary rehabilitation programme consisted of physical training in one of six physiotherapy practices in the Hamburg metropolitan area. All 8 participating physiotherapists were specially trained in respiratory physiotherapy. As a preparation for the study, all physiotherapists were instructed in detail regarding structure and practical application of the training elements. The programme design aimed to allocate subjects to a training location in their neighbourhood to minimise travelling. Subjects were included in the final evaluation if they attended ≥60% of sessions. In addition to exercise training, education on COPD using a validated programme (COBRA [13]) (8 h), nutrition (2 h), psycho-social (2 h) and social (2 h) counselling was offered.

Weekly training sessions were held using only simple training devices such as chairs, elastic bands, sticks, and hand weights. No sophisticated training equipment was used. No physician was present during the training sessions. During the first 8 sessions of 20 minutes duration one physiotherapist taught a single participant training elements and breathing technique. The remaining 18 sessions of 60 minutes duration were held in groups of 6–10. Elements of each session were breathing techniques such as pursed-lip breathing and diaphragmatic exercise, cough technique, progressive muscle relaxation [14], strength, endurance, and coordination training. The structure of training sessions is shown in Table 1. Physiotherapy and endurance training were the main elements of the initial training sessions. Subsequently, increasing emphasis was given to strength and coordination training. Training intensity was guided by the patients’ self-assessment using the Borg scale and was supervised by the attending physiotherapist [15]. Subjects were advised to exercise at a submaximal level, defined as a dyspnoea level of 4–6. Cycle ergometry results were not used to guide training intensity. Exercise training was practised as interval training. In addition to the supervised sessions patients were advised to perform the exercises at home and were given specific training targets.

**Table 1:** Design of training sessions

	**Session 1-3**	**Session 4-6**	**Session 7-9**	**Session 10-x**
Physiotherapy	20 min breathing technique, breathing feedback, cough technique, stretching	15 min breathing technique, breathing feedback, cough technique, stretching	10 min breathing technique, breathing feedback, stretching	10 min breathing technique, breathing feedback, stretching
Endurance	15 min arm/leg exercise against gravity, exercise sitting on a chair: back and abdominal muscles, walk training	15 min arm/leg exercise against gravity, exercise sitting on a chair: back and abdominal muscles, walk training	15 min arm/leg exercise with elastic band, stairs/stepper, walk training	20 min arm/leg exercise with elastic band, stairs/stepper, walk training
Strength	10 min hold against gravity, exercise sitting on a chair, ADL	10 min hold against gravity, exercise sitting on a chair, ADL	15 min exercise sitting on a chair, stairs/stepper, ADL	15 min stairs/stepper ADL elastic band
Coordination	10 min sitting on a chair, standing with/without devices	10 min sitting on a chair, standing with/without devices	15 min in motion with devices	15 min in motion with devices
Home exercise instructions / break	5 min	5 min	5 min	5 min

ADL: activites of daily living; device: stick, towel, elastic band.

#### Sample size

The sample size calculation was based on detecting a between-group difference in 6MWT of 54 m at six months assuming a standard deviation of 80 m. We chose the difference of 54 m as this value was reported to represent the minimal clinically important difference in COPD patients [16]. The standard deviation of 6MWT was estimated as 80 m according to published values in the range of 59 [17] to 92 m [18]. This calculation indicated that 70 subjects (35 per group) were sufficient to provide 80% power to detect a difference of 54 m in 6MWT test at the two-sided 5% level. To allow for a 30% loss to follow-up, 100 participants (50 per group) were considered necessary.

### Statistical analyses

Statistical software (SPSS 10) was used for the analyses. For the primary and secondary outcome measures, the mean results plus 95% confidence intervals (95% CI) were determined using repeated measures analysis with polynomial regression. Differences between groups and before/after the programme were analysed using the unpaired or paired, two-tailed t-test respectively for normally distributed variables, and the Mann–Whitney U-test for non-normally distributed variables. Type I error was set at a level of 0.05 for all statistical analyses.

## Results

### Group characteristics and baseline assessment

**Figure 1:**
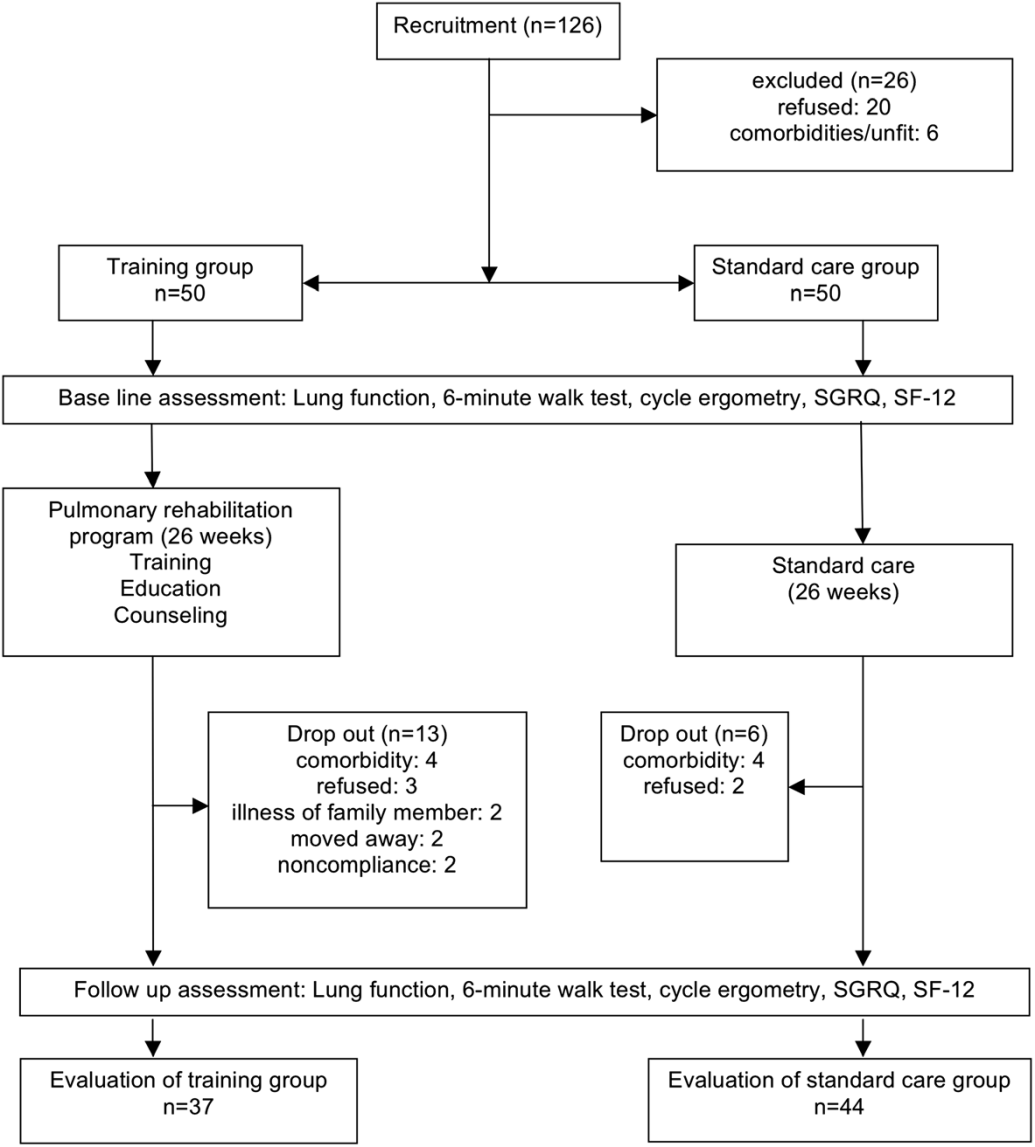
CONSORT flow diagram.

126 subjects were referred from the pulmonary specialist practices for initial screening, see Figure 1 for patient flow. 20 refused to participate in the study and 6 patients were excluded due to relevant comorbidities. A group of 100 subjects with COPD GOLD stage II-IV was randomised. Following randomisation both training and standard care groups consisted of 50 participants.

Descriptive characteristics and baseline assessment results of both groups are shown in Table 2. Randomisation resulted in no significant group differences with the exception of significantly higher SGRQ symptom scores in the control group. 40% were living alone, 24% were working, and 35% were current smokers at the start of the programme. Mean travel distance to the training site was 8.5 ± 6 km.

**Table 2:** Baseline patient characteristics

	**Standard group**	**Training group**
Number	44	37
Gender	24 m / 20 f	23 m / 14 f
Age (years)	65 ± 8	63 ± 11
BMI (kg/m^2^)	25 ± 5	24 ± 5
FEV_1_ (%predicted)	45 ± 13	47 ± 13
ITGV (%predicted)	140 ± 35	150 ± 43
6MWT (m)	429 ± 92	415 ± 83
6MWT (%predicted)	67 ± 13	63 ± 13
Work_max_ (Watt)	75 ± 23	84 ± 30
VO_2_max (l/min)	1.05 ± 0.364	1.05 ± 0.398
CPET time (min)	8.9 ± 2.5	9.4 ± 3.4
Peak heart rate during CPET (1/min)	116 ± 18	121 ± 11
SF-12 PCS	33 ± 9	35 ± 10
SF-12 MCS	52 ± 13	53 ± 12
SGRQ symptoms	55 ± 20	44 ± 23
SGRQ activity	60 ± 20	57 ± 16
SGRQ impact	36 ± 17	31 ± 17
SGRQ total	47 ± 17	41 ± 15

6MWT: 6 minute walk test; BMI: body mass index; CPET: cardiopulmonary exercise testing; FEV_1_: forced expiratory volume in one second; ITGV: intrathoracic gas volume; SF-12 MCS: Short form-12 mental component summary scale score; SF-12 PCS: Short form-12 physical component summary scale score; SGRQ: St. George’s Respiratory Questionaire; VO_2_max: maximum oxygen uptake during cycle ergometry; Work_max_: maximum work rate during cycle ergometry.

There was no statistically significant difference between training and control groups regarding the exacerbation rate: 7/50 vs. 5/50. The dropout rate was 26% (13/50) in the training group and 12% (6/50) in the control group. 2 patients in the training group and 1 patient in the control group dropped out due to a severe exacerbation with hospitalization that precluded the follow-up assessment.

Excluded subjects were not significantly different from the study population in terms of age, sex distribution or lung function. In the control group, 6 subjects could not be evaluated at the follow-up visit. Therefore, 44 subjects in the control group and 37 subjects in the training group completed the study. All analyses refer to these two groups.

### Effects of the pulmonary rehabilitation programme

Parameters of pulmonary function did not change to a clinically meaningful extent during the study period in either group.

Between group changes of the outcome measures over the study period are shown in Table 3. Significant differences were observed regarding 6MWT, maximum work load and SGRQ total score. Furthermore, maximum oxygen uptake and cardiopulmonary exercise testing time differed significantly between groups. There were no adverse events attributable to the training protocol. Average attendance rate in the training group was 75% (range 50-100%). Attendance rates regarding the educational and counseling elements of the programme was 70% in the intervention group.

**Table 3:** **Differences from baseline to6 months between trainingand control group forsix minute walk test,spiroergometry, St. George’s RespiratoryQuestionnaire score, and ShortForm-12 questionnaire score**

	**Complete study population (n** **= 81)**		
	**Between group difference (training minus control group)**	**95% confidence interval**	**p**
6MWT (m)	59	28 to 89	0.0002
Work_peak_ (Watt)	7.4	0.5 to 13.4	0.04
VO_2_peak (l/min)	0.189	0.035 to 0.323	0.02
CPET time (min)	1.7	0.04 to 2.8	0.02
Peak heart rate during CPET (1/min)	4	−2 to 8	n.s.
SF-12 PCS	0.6	−4.1 to 5.2	n.s.
SF-12 MCS	3.0	−3.5 to 9.5	n.s.
SGRQ symptoms	−3	−6 to 12	n.s.
SGRQ activity	−6	−11 to −1	0.03
SGRQ impact	−8	−14 to −2	0.01
SGRQ total	−5	−10 to −1	0.01

6MWT: Six minute walk test; CPET: cardiopulmonary exercise testing; MCS: Mental health component summary score; PCS: Physical component summary score; SF-12: Short form 12 quality of life questionnaire; SGRQ: St. George’s Respiratory Questionnaire.

Table 4 shows intragroup changes during the study period of 6 months. The intervention group increased 6MWT whereas the control group deteriorated compared to baseline. Activity, impact, and total SGRQ scores improved in the intervention group. SF-12 physical component scores increased in both groups during the study period.

**Table 4:** **Differences within groups frombaseline to 6 monthsin six minute walktest, cycle ergometry, St.George’s Respiratory Questionnaire score,and Short Form-12 questionnairescore**

	**Control group (n =** **44)**			**Training group (n =** **37)**		
	**within group difference**	**95% confidence interval**	**p**	**within group difference**	**95% confidence interval**	**p**
6MWT (m)	−21	−42 to −2	0.036	38	22 to 60	0.003
Work_max_ (Watt)	−3.3	−7.8 to 1.2	n.s.	4.1	−0.1 to 8.2	n.s.
VO_2_max (l/min)	−0.029	−0.084 to 0.027	n.s.	0.161	0.010 to 0.311	n.s. (0.059)
CPET time (min)	−0.29	−0.71 to 0.12	n.s.	1.41	0.09 to 2.73	n.s. (0.06)
Peak heart rate during CPET (1/min)	−1	−4 to 1	n.s.	2	−2 to 7	n.s.
SF-12 PCS	4.2	1.2 to 7.3	0.03	4.8	1.5 to 8.0	0.006
SF-12 MCS	−0.6	−5.0 to 3.9	n.s.	2.7	−0.7 to 6.2	n.s.
SGRQ symptoms	−5	−11 to 1	n.s.	−2	−8 to 4	n.s.
SGRQ activity	0	−3 to 4	n.s.	−5	−9 to −1	0.02
SGRQ impact	−1	−4 to 2	n.s.	−9	−14 to −4	0.001
SGRQ total	−1	−4 to 1	n.s.	−7	−10 to −3	0.001

6MWT: Six minute walk test; CPET: cardiopulmonary exercise testing; MCS: Mental health component summary score; PCS: Physical component summary score; SF-12: Short form 12 quality of life questionnaire; SGRQ: St. George’s Respiratory Questionnaire; VO_2_max: maximum oxygen uptake during cycle ergometry; Work_max_: maximum work rate during cycle ergometry.

### Costs

Staff costs of the program for the physiotherapists were €469 (group size of 10) to €625 (group size of 6) per patient for 6 months. The personnel cost of each training session was €53 which represents the hourly salary of the physiotherapist. As no training machines or cycle ergometers were used, investments to cover infrastructure were reduced to a minimum. To calculate the overall cost of the program renting cost for rooms, taxes, secretarial expenses and expenditure for diagnostic procedures need to be added. A minimum group size of six resulted in staff cost per training session and participant of €9. Hence, staff cost for a 6 months extension of training sessions sum up to €234. Parts of the training session costs were paid by the participants (€5 per session). The remaining costs were covered by the unrestricted educational grant from the sponsor of the study.

## Discussion

Our study in patients with moderate to severe COPD shows that a physiotherapist-lead, long-term pulmonary rehabilitation programme of lower training intensity and frequency than currently recommended achieved clinically significant improvements in terms of physical capabilities and HRQoL. The observed results fell above previously published thresholds for minimal clinically important differences regarding 6MWT (54 m), maximum work load (4 ± 1 Watt) and SGRQ total score (4 points) [19][20][21]. The effect sizes were comparable to results of previously published programmes using higher intensity and costs [16-18,22,23].

However, the intervention used in our study differs from previous studies. Most long-term studies evaluated maintenance programmes following an initial intense rehabilitation programme [22-24]. In contrast, our programme consisted of low intensity weekly training over the whole study interval, which is deemed insufficient by the ATS/ERS guidelines [1].

Troosters et al. in their pioneer study evaluated a long-term outpatient rehabilitation programme and found similar effects in a population comparable to ours [16]. However, their programme required a significantly greater amount of resources, namely personell and equipment. In addition, training was performed two to three times a week in a single specialized center.

Guell et al. addressed long-term effects of outpatient rehabilitation in COPD [17]. The impressive improvements in 6MWT are difficult to interpret, as the investigators did not report practice tests and whether assessors were blinded. Furthermore, improvements were obtained during the first 3 months while only breathing retraining and no exercise training was performed.

Recently, a physiotherapist-lead programme similar to ours was reported to induce favourable effects regarding exercise capabilities and subdomains of HRQoL questionaires [25]. Again, the frequency was higher (2–3 sessions per week) and the group size smaller than in our study (2–3 participants).

The question arises why our programme achieved the presented results despite using a training intensity, frequency and total time that was lower than that recommended by current guidelines [1] on which most current rehabiliation programmes were based. It is well known, that in the short-term, programmes of higher intensity and frequency produce better results in terms of exercise capabilities [1]. However, in long-term programmes factors other than these appear to be crucial for success.

First, it appears obvious that the overall amount of home training rather than supervised training time determines the efficacy of long-term pulmonary rehabilitation. As yet, studies measuring the amount of home training with sufficient precision are lacking. A certain frequency of supervised training sessions appears to be necessary as a reminder to maintain increased physical activity at home. Repeated courses, telephone interventions, and regularly scheduled visits of medical personell have been shown to have only modest impact on long-term outcomes [1]. Our study shows that weekly sessions may represent a sufficient stimulus for unsupervised exercise to induce significant effects. In line with our observation, Spencer et al. reported successful maintenance for 12 months of initially achieved improvements by either once weekly supervised or even unsupervised home training [23]. Interestingly, the American College of Sports Medicine published guidelines for older adults and people with chronic diseases, which reduced the minimum recommended level of effective physical exercise [26].

Second, reaching the highest intensity of supervised training is of lesser importance in the long-term setting. If severely impaired patients cannot follow the training protocol due to excessive demands its effects and consequently patient adherence will be significantly reduced [27]. These findings stress the point that in terms of long-term efficacy it appears to be more important to find an adequate, i.e. tolerable, level of training intensity rather than aiming at the highest possible intensity. In line with these results, the ATS/ERS guidelines state that while high-intensity targets may be beneficial for inducing physiological changes in patients who can reach these levels, low intensity targets may be more appropriate to achieve long-term adherence and health benefits for a wider population [1].

Third, adherence is of significant importance. A recent study found that with adherence rates below 70% no improvement can be expected from rehabilitation programmes [22]. In our programme adherence was 75%. We speculate that offering training sites in the patients’ neighbourhood was of critical importance for the success of our programme as travel time is a well known reason for non-adherence [28]. Patient-perceived barriers such as excessive intensity, costs or distance to training site were recently found to be predictive of a failure to maintain initially achieved rehabilitation effects [29].

The practicability of any rehabilitation programme depends on the average cost and local access for a wide patient population. In a recent review, the affordability of the average costs of $2615 per patient per 6 months reported in the pilot study of Troosters was questioned in current health care systems [30]. Poor access to pulmonary rehabilitation programmes impedes widespread use of this effective intervention [2]. To increase accessability, we designed our programme using the locally available infrastructure, i.e. supervised training was performed in physiotherapy practices. This design and the fact that only once weekly supervised sessions were held resulted in reduced costs per patient, which appeared to be lower than in previously published programmes. Obviously, the presented cost calculation needs to be interpreted with caution as it is difficult to transfer into other health care settings.

The observed dropout rate of 26% is in the previously reported range from 19 to 31% [16,23,24]. This documents that our programme was feasible under conditions close to real life. Therefore, we expect that the effects of our programme may be repeated on a broader scale.

The present study has some limitiations. As we did not incorporate an intervention group using higher training intensity and frequency, we cannot rule out that an intervention conforming more closely to the guidelines might have induced greater effects. However, as the effects detected in our study fall in the upper range of published results, we doubt that an increase in intensity and/or frequency would have produced a significant additional effect.

We only performed outcome measurements after baseline assessments. Thereby, we do not know whether there was an initial improvement that vanished subsequently as has been reported from effects achieved by short-term programmes. However, it has been consistently reported that rehabilitation effects might be sustained as long as the intervention is continued [16,17,22-24].

We did not measure the actual extent of overall physical activity. Hence, we can only speculate that life style changes, i.e. increased physical activity in daily life, induced the observed effects, as Effing et al. detected using pedometers in a study similar to our programme [25]. However, as long as we do not have data regarding physical activity at home, further studies extending the findings of our pilot study are needed before it can be stated that a programme of lower intensity is equally effective as currently recommended programmes.

6MWT assessors were not blinded to allocation status, possibly causing a bias. We do not believe that this factor influenced our results, as changes in 6MWT compare well to those seen during cycle ergometry, which was performed in a blinded manner.

We did not use the results of cardiopulmonary exercise testing to adjust training intensity as it usually done. Instead, training intensity was guided by dyspnea ratings using the Borg scale. It has been shown before that dyspnea ratings and/or heart rate measurements can be used as a target for patients with COPD to regulate/monitor the intensity of exercise training [31,32].

In contrast to SGRQ activity, impact and total scores, which significantly improved in the training group compared to the control group, SGRQ symptom scores showed a reverse pattern with greater improvements seen in the control group. This observation may be purely by chance, but may also point to the aspect that exercise training does not alter the disease itself with its symptoms of cough and sputum production.

In summary, this study shows that a long-term, physiotherapist-lead pulmonary rehabilitation programme using a lower training intensity and frequency than currently recommended may achieve clinically relevant improvements in exercise capabilities and health-related quality of life in patients with moderate to severe COPD. However, before low-intensity programmes as ours may be rated as comparably effective as currently recommended programmes, further studies corroborating our findings are needed. The simple design and small amount of necessary resources may help to offer effective pulmonary rehabilitation to a greater proportion of eligible patients. Results may only be reproducible in a similar setting with adequately motivated patients.

## Competing interests

All authors declare that they have no competing interests.

## Authors’ contributions

HJB, TS, and AM participated in developing the study design, KR collected the data, KR and HJB performed the statistical analyses, HJB and AM drafted the manuscript. All authors revised the manuscript critically for important intellectual content. All authors read and approved the final manuscript.
